# Are women with type 2 diabetes mellitus more susceptible to cardiovascular complications following coronary angioplasty?: a meta-analysis

**DOI:** 10.1186/s12872-017-0645-8

**Published:** 2017-07-27

**Authors:** Pravesh Kumar Bundhun, Manish Pursun, Feng Huang

**Affiliations:** 1 Institute of Cardiovascular Diseases, the First Affiliated Hospital of Guangxi Medical University, Nanning, Guangxi 530027 People’s Republic of China; 2 Guangxi Medical University, Nanning, Guangxi 530027 People’s Republic of China

**Keywords:** Type 2 diabetes mellitus, Percutaneous coronary intervention, Gender, Female sex, Major adverse cardiac events, Mortality

## Abstract

**Background:**

Scientific reports have shown Type 2 Diabetes Mellitus (T2DM) to be independently associated with adverse outcomes following Percutaneous Coronary Intervention (PCI). However, gender difference has also often been a controversial issue following PCI. Till date, very few meta-analyses have systematically compared the adverse cardiovascular outcomes in male versus female patients with T2DM following PCI. Therefore, we aimed to carry out this analysis in order to find out an answer to this interesting question.

**Methods:**

Electronic databases were searched for English language publications reporting adverse cardiovascular outcomes in male versus female patients with diabetes mellitus respectively following coronary angioplasty. The RevMan 5.3 software was used to analyze selected adverse cardiovascular events whereby Odds Ratios (OR) and 95% Confidence Intervals (CI) were the statistical parameters.

**Results:**

A total number of 19,304 patients with T2DM (12,986 male patients versus 6318 female patients) were included in this analysis. At baseline, female patients were older (68.7 versus 62.9 years), with a higher percentage of hypertension (75.6% versus 66.5%) and dyslipidemia (53.3% versus 50.0%) whereas majority of the male patients were smokers (46.3% versus 14.9%). Results of this analysis showed short and long-term mortality to be significantly higher in female patients with T2DM (OR: 1.71, 95% CI: 1.46–2.00; *P* = 0.00001), and (OR: 1.20, 95% CI: 1.07–1.35; *P* = 0.002) respectively. In addition, women were also more at risk for short and long-term major adverse cardiac events (MACEs) with OR: 1.49, 95% CI: 1.07–2.07; *P* = 0.02 and OR: 1.15, 95% CI: 1.04–1.28; *P* = 0.009 respectively. Subgroup analysis showed this significant result to have mainly been observed in patients with acute myocardial infarction compared to those with stable coronary artery disease.

**Conclusions:**

Following PCI, women with T2DM were indeed more susceptible to short and long-term cardiovascular complications compared to male patients with the same chronic disease. Even though this result was more applicable to patients with acute myocardial infarction, the fact that women were older with higher co-morbidities at baseline compared to men, should also not be ignored.

## Background

With the unhealthy lifestyle adopted by people nowadays, the population of patients with Type 2 Diabetes Mellitus (T2DM) is expected to rise massively in the coming years [1]. Scientific reports have shown T2DM to be independently associated with adverse outcomes following Percutaneous Coronary Intervention (PCI) [2]. However, when gender is to be taken into account, it is still unclear who among men and women, are at higher risks for complications [3, 4].

In a recent meta-analysis dealing with the geographical difference of the interaction of sex with treatment strategy in patients with multi-vessel disease and left main disease, and involving the SYNTAX (Synergy Between PCI With Taxus and Cardiac Surgery), PRECOMBAT (Bypass Surgery Versus Angioplasty Using Sirolimus Eluting Stent in Patients With Left Main Coronary Artery Disease), and BEST (Bypass Surgery and Everolimus Eluting Stent Implantation in the Treatment of Patients With Multivessel Coronary Artery Disease) Randomized Controlled Trials, the authors suggested that the heterogeneity of gender-treatment interaction needed to be well-recognized and considered when deciding the treatment strategy [5].

However, till date, only few meta-analyses have systematically compared adverse cardiovascular outcomes between men and women with T2DM following PCI. Therefore, for a better management of diabetic male and female patients with cardiovascular diseases, we aimed to carry out this analysis in order to find an answer to this interesting question.

## Methods

### Searched databases and strategies

Electronic databases (PubMed, EMBASE and the Cochrane Central Register of Controlled Trials) were searched for English language publications reporting adverse cardiovascular outcomes in male versus female patients with diabetes mellitus following coronary angioplasty.

During this search process, the terms ‘diabetes mellitus, gender and percutaneous coronary intervention’ were used. The words ‘diabetes mellitus’ were also replaced by the abbreviation ‘T2DM’, the term ‘gender’ was also replaced by the word ‘sex, women or men’ respectively whereas the words ‘percutaneous coronary intervention’ were also replaced by the term ‘coronary angioplasty’ or the abbreviation ‘PCI’.

Reference lists of qualified publications were also reviewed for any relevant article. In addition, official websites of major cardiovascular related journals such as the Journal of American College of Cardiology, Journal of Circulation, and Cardiovascular Diabetology were also checked for relevant articles.

### Inclusion and exclusion criteria

Studies were included if they were trials or observational studies comparing adverse clinical outcomes between male and female patients with T2DM following PCI. In addition, studies which dealt with the general population, but consisted of more than 30% of female patients with T2DM were also qualified for this analysis.

Studies were excluded if:

- They were meta-analyses, case studies or letter to editors.
- They did not include patients with T2DM.
- They did not compare male with female patients.
- They did not report adverse cardiovascular outcomes following PCI.
- They were duplicates.

### Type of participants, outcomes and follow ups

This analysis included patients with stable coronary artery disease (CAD), acute myocardial infarction (AMI), and specifically patients with STEMI as shown in Table 1.

**Table 1:** Type of participants, reported outcomes and follow-up periods

Studies	Outcomes	Follow-up periods	Type of patients
Blondal 2012 [9]	MACEs, MI, revascularization, death	2.1 years	AMI
Buja 2012 [26]	MACEs, death, MI, TVR, TLR	24.3 months	CAD
Holland 2013 [12]	Death, MACEs, revascularization	5 years	CAD
Lin 2013 [27]	Death	5.4 years	CAD
Michelle 2003 [25]	Death	1 year	CAD
Younan 2015 [28]	Death, MI, TVR, MACEs	1 year	CAD
Epps 2016 [29]	MACEs, death, MI, revascularization, TLR, TVR	In-hospital, 1 and 5 years	CAD
Toyota 2013 [30]	Death, MACEs, TLR, revascularization	In-hospital and 3 years	AMI
Cheng 2004 [31]	Death	30 days	AMI
Eitel 2011 [32]	Death	30 days	STEMI
Kosuge 2006 [33]	Death, MI	In-hospital	AMI
Liu 2015 [11]	Death, MI	30 days	AMI
Qi 2010 [34]	Death, MI, MACEs, TLR	30 days	STEMI
Roffi 2013 [10]	Death, MI	30 days	STEMI
Zanchi 2009 [35]	Death	In-hospital	STEMI

*Abbreviations*: *MACEs* major adverse cardiac events; *MI* myocardial infarction; *TVR* target vessel revascularization; *AMI* acute myocardial infarction; *CAD* coronary artery disease; *STEMI* ST segment elevated myocardial infarction

The outcomes which were analyzed [death, myocardial infarction (MI), repeated revascularization, major adverse cardiac events (MACEs)] have been listed in Table 1.

Death referred to all-cause mortality whereas MACEs consisted mainly of death, MI and revascularization or stroke.

A short-term follow up (less or equal to 30 days) and a longer follow up period (> one year) were considered relevant in this analysis.

### Data extraction and review

The following data were independently extracted by two reviewers (PKB and MP):

- Names of authors
- Year of publication
- Type of study (observational or randomized trial)
- Number of male patients
- Number of female patients
- Patients’ enrollment periods
- Baseline features
- Outcomes which were assessed
- Follow-up periods
- Adverse events which were reported in male versus female patients respectively

Disagreements were resolved by a thorough discussion with the third author (FH).

The PRISMA guideline [6] was followed, and the bias risk was assessed as advised by the Cochrane Collaboration [7], for the only trial which was included.

### Statistical analysis

The RevMan software version 5.3 was used to analyze selected adverse cardiovascular events which were reported between male and female patients with T2DM following PCI, whereby odds ratios (OR) and 95% confidence intervals (CI) were used as the statistical parameters.

Heterogeneity [8] across the subgroups analyzing the respective outcomes was assessed using the:

(i) Cochrane Q statistic test (*P* ≤ 0.05 was considered statistically significant).
(ii) I^2^ statistic test (a low value of I^2^ represented a low heterogeneity. To be more specific, an I^2^ value below 25% represented a lower heterogeneity, an I^2^ value about 50% represented a moderate heterogeneity whereas an I^2^ value approaching 100% (75–100%), represented a high heterogeneity).

In this analysis, a fixed (I^2^ < 50%) or a random (I^2^ > 50%) effects model was used depending on the I^2^ value which was obtained.

Since this analysis involved a low volume of studies, publication bias was visually estimated by assessing funnel plots which were directly generated by the RevMan 5.3 software.

Sensitivity analysis was also carried out (excluding each study one by one and then carrying out a new analysis each time to observe any significant difference in the results).

### Ethics and patients’ consents

Ethical approval and patients’ consents were not applicable.

## Results

### Searched outcomes

**Fig. 1:**
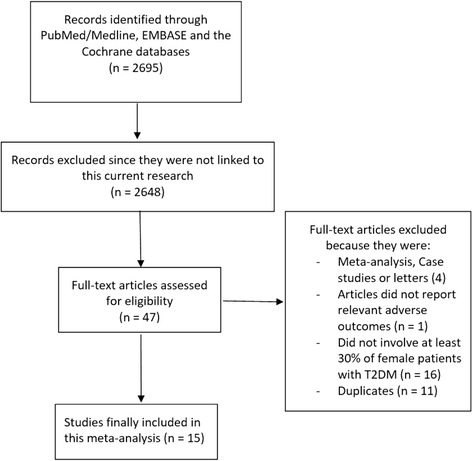
Flow diagram representing the study selection

A thorough search from electronic databases ended with a total number of 2695 articles. Following an assessment of the titles and abstracts, 2648 articles were eliminated since they were not linked to this current research. Forty-seven (47) full text articles were assessed for eligibility. However, after a careful assessment of these articles, further publications were eliminated since: they were case studies (3), meta-analysis (1), they did not report adverse clinical outcomes (1), they compared male versus female patients following PCI but, however, they consisted of less than 30% of women with T2DM (16), they were duplicates (11). Finally, 15 articles were confirmed for this analysis (Fig. 1).

### General features of the studies which were included

Only one randomized trial was included, whereas the other studies were observational studies. This analysis consisted of a total number of 19,304 patients (12,986 male patients and 6318 female patients). Patients’ enrollment period ranged from the year 1993 to 2014 (Table 2). Following the bias risk assessment, a grade B was given to the only randomized trial which was included in this analysis.

**Table 2:** General features of the studies which were included

Studies	Patients’ enrollment (years)	No of male patients (n)	No of female patients (n)	Type of study
Blondal 2012 [9]	2006–2009	155	142	Observational
Buja 2012 [26]	2003–2009	1741	679	Observational
Holland 2013 [12]	2001–2005	1666	702	RCT
Lin 2013 [27]	1997–2003	239	147	Observational
Michelle 2003 [25]	1995–1998	2411	1074	Observational
Younan 2015 [28]	2010–2013	100	100	Observational
Epps 2016 [29]	1997–2006	1699	1303	Observational
Toyota 2013 [30]	2005–2007	1046	380	Observational
Cheng 2004 [31]	1993–2000	199	61	Observational
Eitel 2011 [32]	-	56	36	-
Kosuge 2006 [33]	2001–2003	672	273	Observational
Liu 2015 [11]	2010–2014	117	52	Observational
Qi 2010 [34]	2005–2008	348	171	Observational
Roffi 2013 [10]	1997–2010	2412	1153	Observational
Zanchi 2009 [35]	2004–2008	125	45	Observational
Total no of patients (n)		12,986	6318	

*Abbreviations*: *RCT* randomized controlled trials

### Baseline features of the male and female patients

**Fig. 2:**
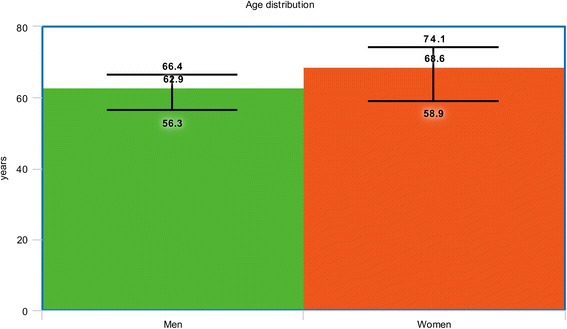
Graphical representation of age between men and women at baseline

The baseline characteristics of the male and female patients with T2DM have been summarized in Table 3. Male patients had a mean age varying between 56.3 and 66.4 years whereas female patients had a mean age ranging from 58.9 to 74.1 years. Figure 2 shows a graphical representation of age between male and female patients at baseline.

**Table 3:** Baseline features of the patients

Studies	Age (yrs)	Ht (%)	Ds (%)	Cs (%)	PCI* (%)	CABG* (%)
	M/F	M/F	M/F	M/F	M/F	M/F
Blondal 2012 [9]	65.0/69.3	80.7/91.6	62.5/69.7	30.3/7.00	20.7/9.20	3.90/3.40
Buja 2012 [26]	64.9/68.9	73.2/82.5	65.9/69.5	52.1/16.5	11.4/12.2	-
Holland 2013 [12]	62.2/62.9	80.6/87.1	-	13.4/10.3	-	-
Lin 2013 [27]	63.4/66.5	74.5/82.3	38.5/46.9	42.3/11.6	53.1/47.9	25.1/29.9
Michelle 2003 [25]	63.6/65.7	61.8/73.6	46.8/44.5	-	12.6/12.1	10.5/7.00
Younan 2015 [28]	57.9/58.9	64.0/56.0	62.0/60.0	45.0/3.00	22.0/18.0	2.00/2.00
Epps 2016 [29]	65.0/69.0	69.4/79.0	70.4/70.4	21.7/19.1	33.8/28.7	22.0/15.4
Toyota 2013 [30]	64.5/74.1	76.7/81.0	-	51.9/14.6	-	-
Cheng 2004 [31]	61.0/67.0	45.0/67.1	42.4/40.8	65.2/5.1	-	0.0/0.0
Eitel 2011 [32]	64.0/72.0	68.0/76.0	33.0/30.0	43.0/34.0	-	-
Kosuge 2006 [33]	65.0/73.0	52.0/64.0	33.0/37.0	57.0/17.0	-	-
Liu 2015 [11]	56.3/69.5	74.4/71.2	45.3/69.2	73.5/13.5	22.2/7.70	-
Qi 2010 [34]	63.9/71.7	51.0/68.8	39.6/44.9	65.6/9.80	5.50/4.10	-
Roffi 2013 [10]	66.4/73.0	70.7/79.4	62.8/61.7	36.3/19.7	-	-
Zanchi 2009 [35]	60.3/67.3	55.7/75.0	47.5/48.4	51.4/27.4	-	5.20/2.40

*Abbreviations*: *yrs.* years; *Ht* hypertension; *Ds* dyslipidemia; *Cs* current smoker; *PCI* percutaneous coronary intervention; *CABG* coronary artery bypass surgery; *M* males; *F* females

*signified previous PCI or previous CABG

**Fig. 3:**
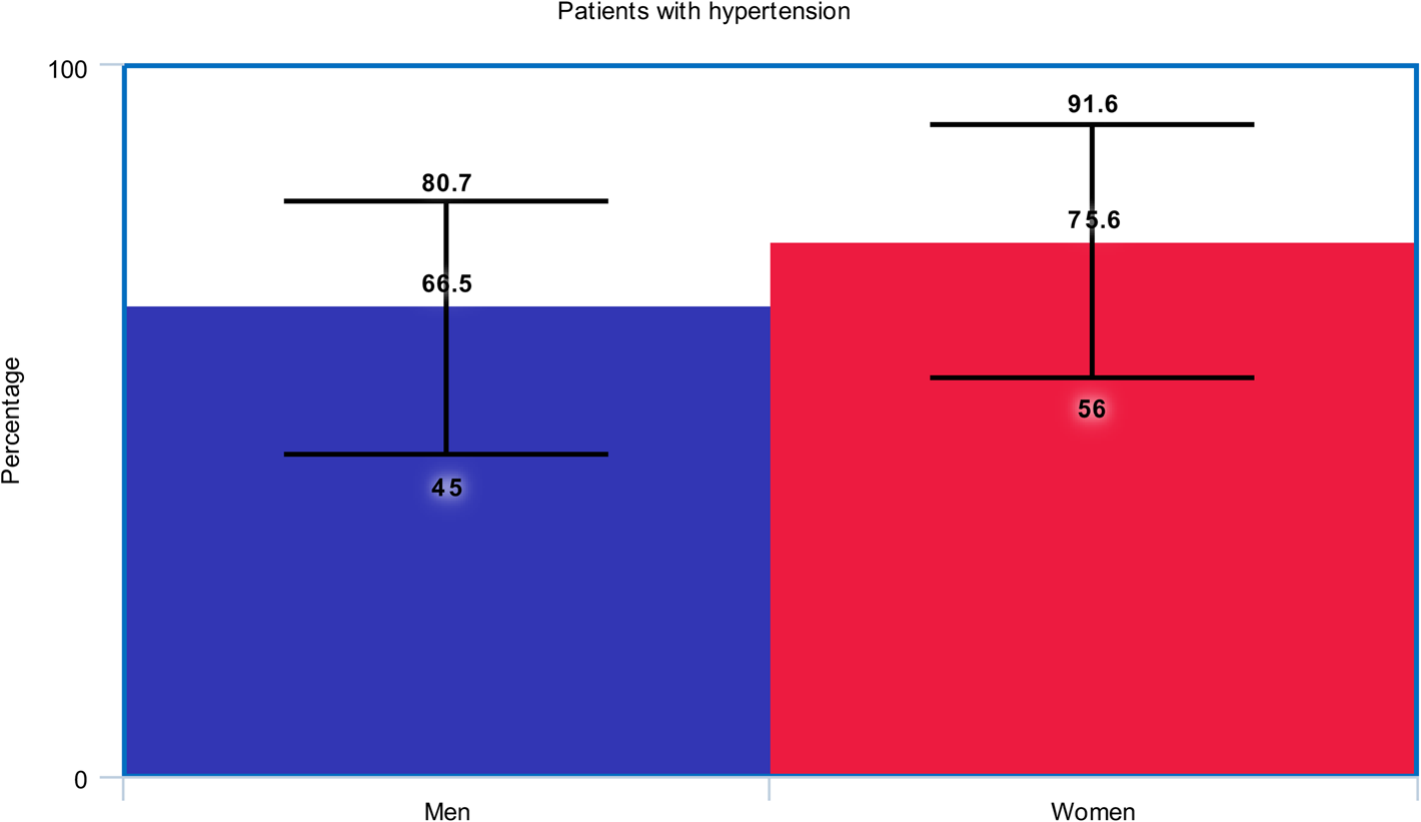
Graphical representation of hypertension between men and women at baseline

**Fig. 4:**
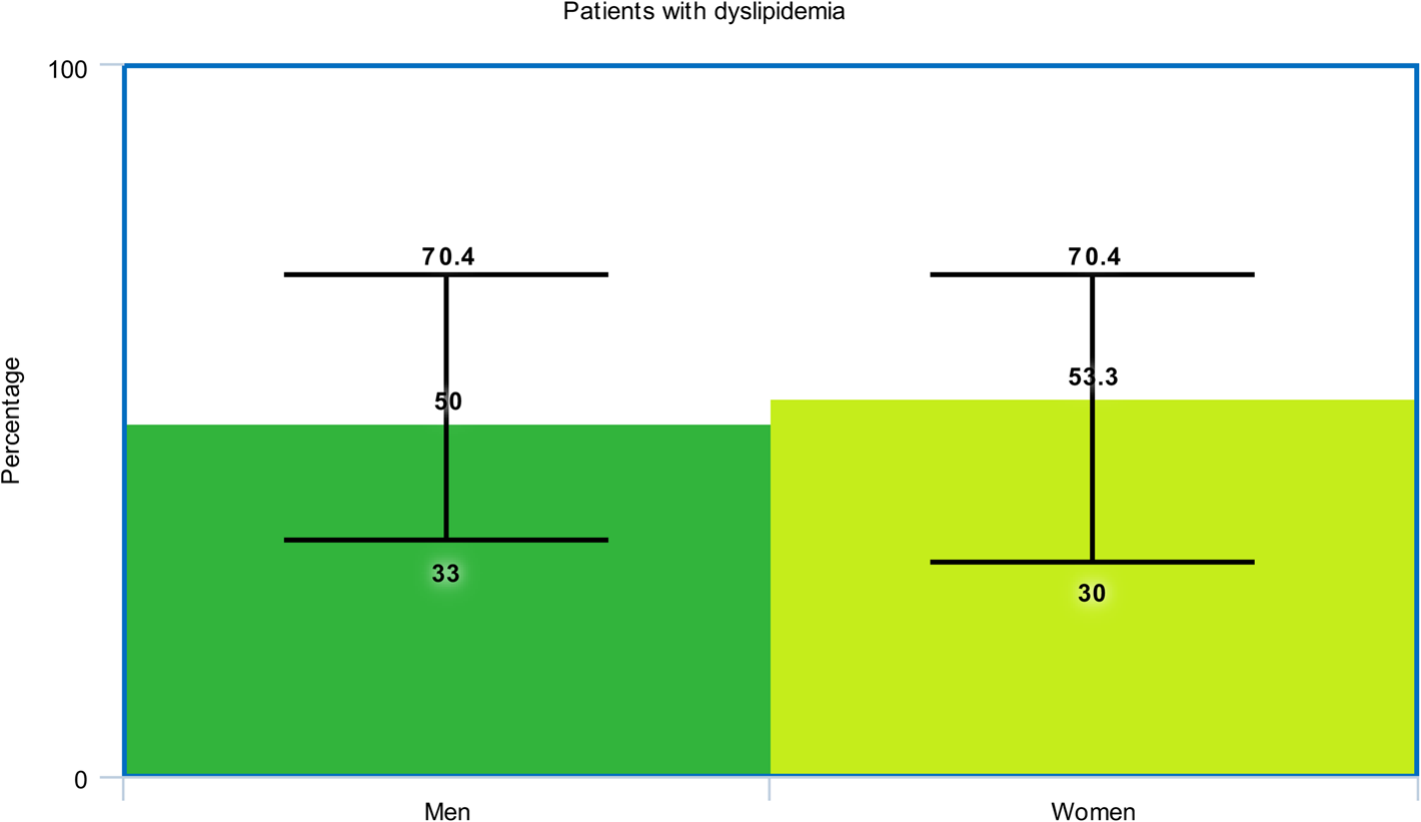
Graphical representation of dyslipidemia between men and women at baseline

The percentage of female patients suffering from hypertension and dyslipidemia were higher with the exception of one or two studies. On average, a graphical representation of hypertension and dyslipidemia has been shown (Figs. 3 and 4).

**Fig. 5:**
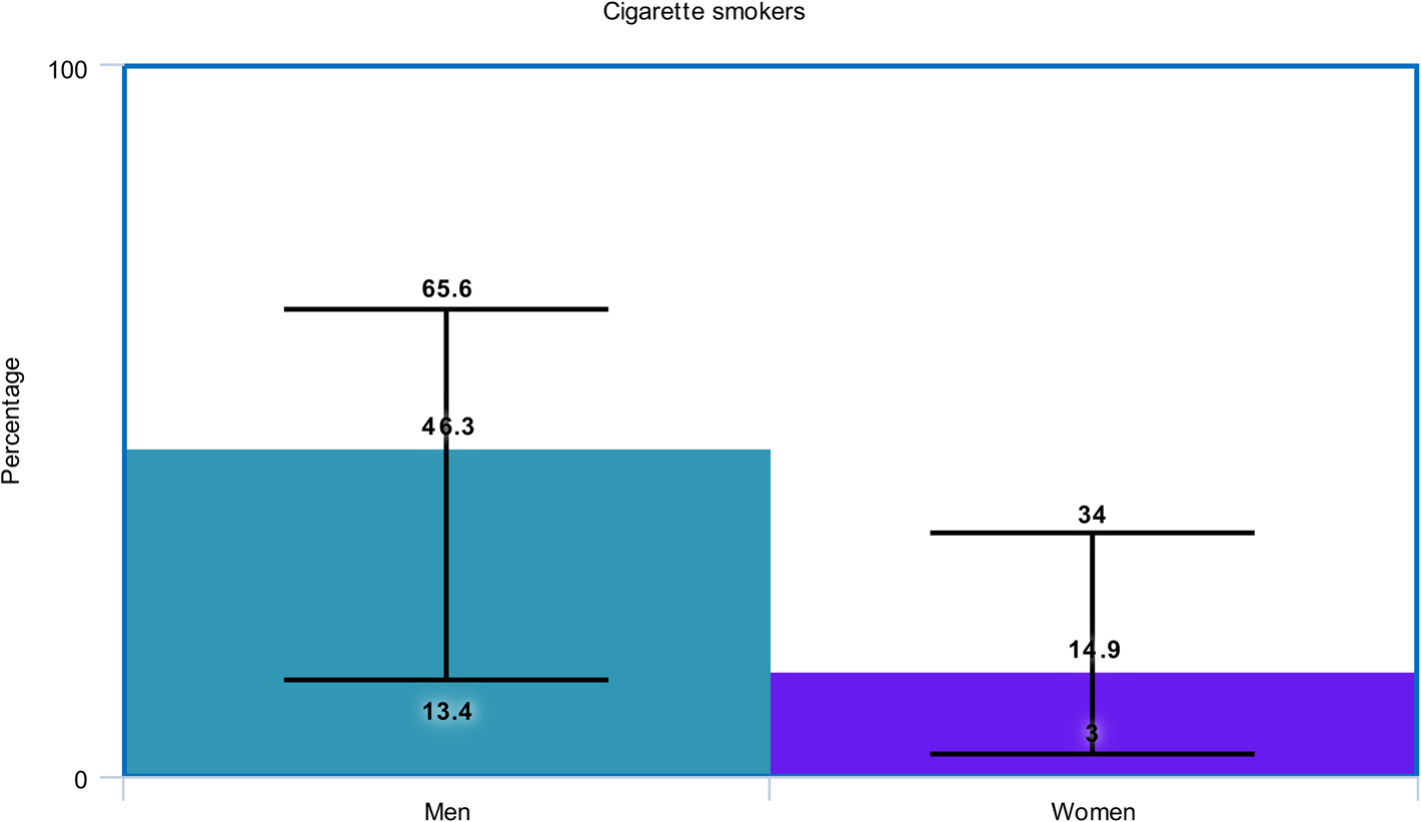
Graphical representation of smokers between men and women at baseline

At baseline, majority of male patients were smokers, compared to female patients as shown in Fig. 5. To summarize the baseline features, female patients were older, and had a higher risk for co-morbidities whereas male patients were heavy smokers.

The medication use at baseline (if reported) has also been summarized in Table 4.

**Table 4:** Medication profile at baseline

Studies	Insulin therapy	Aspirin	Clopidogrel	Statin	Beta-blocker
	Male/female	Male/female	Male/female	Male/female	Male/female
Blondal 2012 [9]	-	99.4/96.5	96.8/90.9	76.8/73.9	82.6/76.8
Buja 2012 [26]	30.6/40.7	-	-	-	-
Holland 2013 [12]	-	91.0/88.0	91.0/88.0	76.0/73.0	72.0/75.0
Lin 2013 [27]	-	-	-	17.6/19.7	43.5/40.1
Michelle 2003 [25]	-	-	-	-	-
Younan 2015 [28]	72.0/68.0	-	-	41.0/39.0	34.0/38.0
Epps 2016 [29]	-	96.0/94.4	-	68.5/65.9	77.0/75.6
Toyota 2013 [30]	4.10/4.70	12.6/13.5	7.00/13.0	11.4/18.4	-
Cheng 2004 [31]	-	-	-	-	-
Eitel 2011 [32]	-	100/98.0	100/98.0	98.0/99.0	99.0/97.0
Kosuge 2006 [33]	-	-	-	-	-
Liu 2015 [11]	-	-	-	-	-
Qi 2010 [34]	-	100/100	100/100	91.7/91.2	71.6/68.6
Roffi 2013 [10]	-	-	-	-	-
Zanchi 2009 [35]	-	-	-	-	-

Data have been reported in percentage

### Short-term adverse outcomes observed between men versus women with T2DM

The main results have been summarized in Table 5.

**Table 5:** Results of this analysis

Outcomes analyzed	No of studies involved (n)	OR with 95% CI	*P* value	I^2^ (%)
Short term outcomes				
Mortality	9	1.71 [1.46–2.00]	0.00001	0
MI	5	1.26 [0.77–2.07]	0.36	63
MACEs	2	1.49 [1.07–2.07]	0.02	37
Long-term outcomes				
Mortality	8	1.20 [1.07–1.35]	0.002	41
MI	4	1.20 [0.95–1.53]	0.13	0
MACEs	6	1.15 [1.04–1.28]	0.009	43
RR	6	0.93 [0.76–1.13]	0.46	56

*Abbreviations*: *MI* myocardial infarction; *MACEs* major adverse cardiac events; *OR* odds ratios; *CI* confidence intervals; *RR* repeated revascularization

**Fig. 6:**
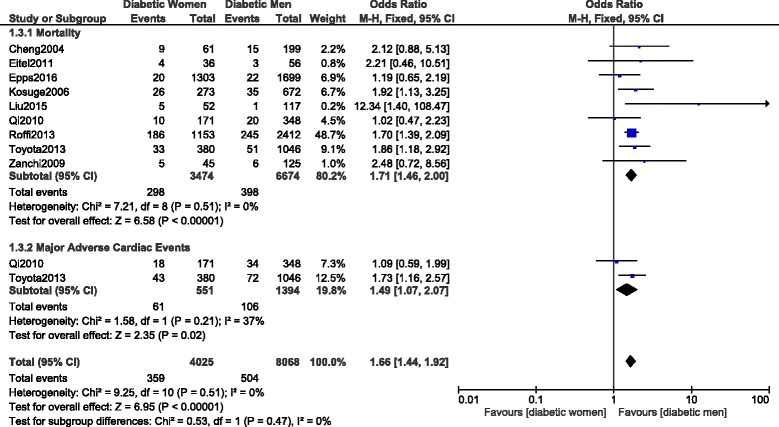
Short-term mortality and MACEs observed between women versus men with T2DM following PCI

Results of this analysis showed that during a short-term follow up period, mortality and MACEs were significantly higher in women with T2DM (OR: 1.71, 95% CI: 1.46–2.00; *P* = 0.00001, I^2^ = 0%), and (OR: 1.49, 95% CI: 1.07–2.07; *P* = 0.02, I^2^ = 37%) respectively as shown in Fig. 6.

**Fig. 7:**
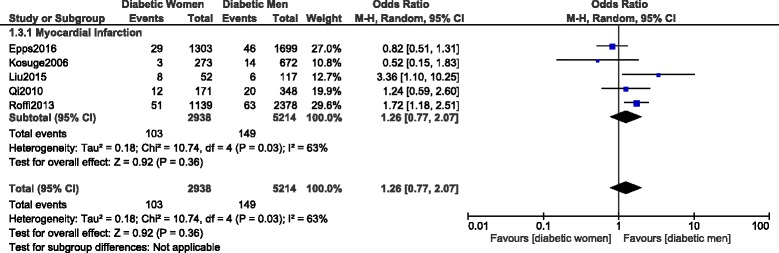
Short-term myocardial infarction observed between women versus men with T2DM following PCI

However, even if short-term MI was also higher in these women (OR: 1.26, 95% CI: 0.77–2.07; *P* = 0.36, I^2^ = 63%), the result was not statistically significant as shown in Fig. 7.

**Fig. 8:**
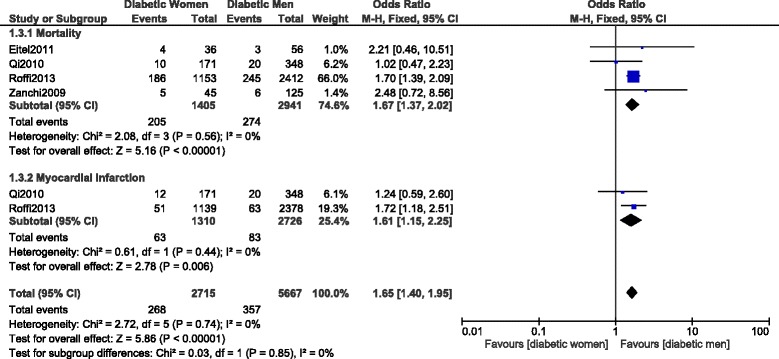
Short-term mortality and myocardial infarction observed in a population of diabetic female versus male patients with STEMI following PCI

Another subgroup analysis was carried out only with STEMI patients. Mortality and MI were significantly higher with women following PCI with OR: 1.67, 95% CI: 1.37–2.02; *P* = 0.00001, I^2^ = 0% and OR: 1.61, 95% CI: 1.15–2.25; *P* = 0.006, I^2^ = 0% respectively (Fig. 8).

### Long-term adverse outcomes observed between men versus women with T2DM

**Fig. 9:**
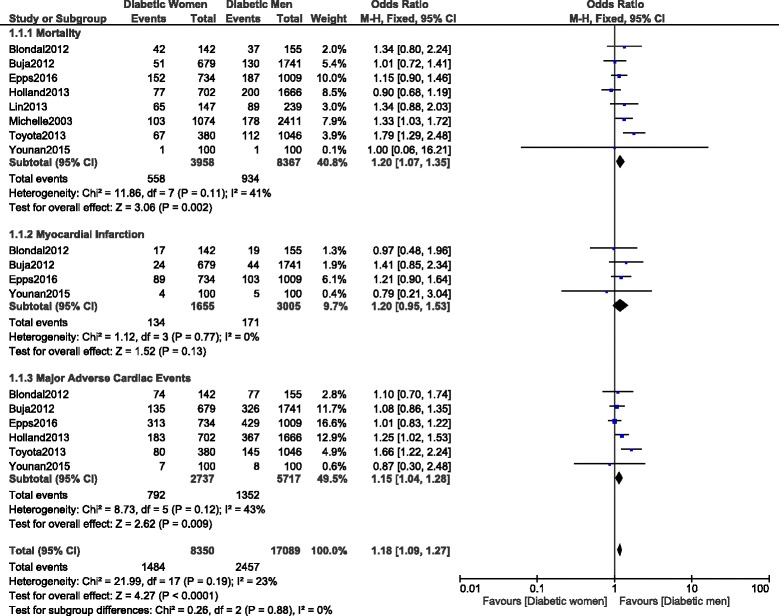
Long-term adverse cardiovascular outcomes observed between women versus men with T2DM following PCI

When the adverse outcomes were analyzed during a longer follow-up period, mortality and MACEs were still significantly higher in female patients with T2DM, with OR: 1.20, 95% CI: 1.07–1.35; *P* = 0.002 and OR: 1.15, 95% CI: 1.04–1.28; *P* = 0.009 respectively (Fig. 9). However, even if MI was also higher in female patients with OR: 1.20, 95% CI: 0.95–1.53; *P* = 0.13, the result was not significant (Fig. 9).

**Fig. 10:**
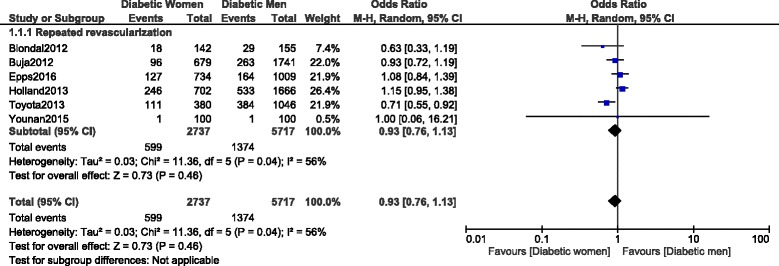
Long-term repeated revascularization observed between diabetic women versus men following PCI

Long-term repeated revascularization was similarly manifested between male and female patients with T2DM, with OR: 0.93, 95% CI: 0.76–1.13; *P* = 0.46 as shown in Fig. 10.

**Fig. 11:**
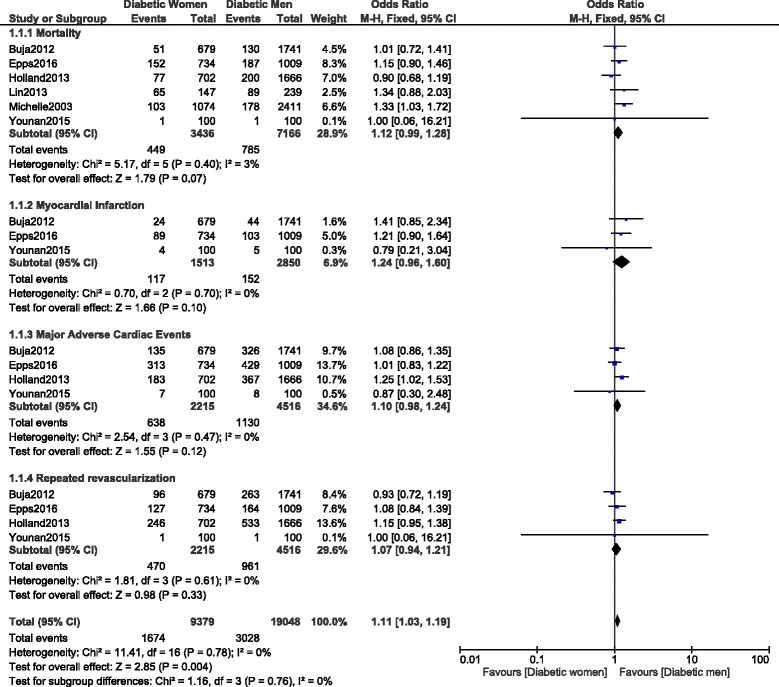
Long-term adverse cardiovascular outcomes observed between diabetic women versus diabetic men with stable coronary artery disease following PCI

Another subgroup analysis was carried out only including diabetic patients with stable coronary artery disease. Even if a higher mortality, MI, MACEs and repeated revascularization were observed in women, with OR: 1.12, 95% CI: 0.99–1.28; *P* = 0.07, I^2^ = 3%, OR: 1.24, 95% CI: 0.96–1.60; *P* = 0.10, I^2^ = 0.10, OR: 1.10, 95% CI: 0.98–1.24; *P* = 0.12, I^2^ = 0% and OR: 1.07, 95% CI: 0.94–1.21; *P* = 0.33, I^2^ = 0% respectively, the results were not statistically significant as shown in Fig. 11.

### Sensitivity analysis

Sensitivity analyses were carried out by excluding each study one by one, and carrying out a new analysis. Sensitivity analysis which was carried out on the subgroup assessing for short-term mortality did not show any deviation from the main result of this analysis. However, for the subgroup analyzing MI, excluding study Epps2016 showed a significantly higher MI in women with T2DM, with OR: 1.56, 95% CI: 1.15–2.11; *P* = 0.005, I^2^ = 44%.

Sensitivity analysis which was carried out on the subgroup assessing for long-term mortality did not show any significant difference compared to the main result. However, in the subgroup assessing for MACEs, excluding study Holland2013 and Toyota2013 respectively, showed a non-significant result with OR: 1.12, 95% CI: 0.99–1.27; *P* = 0.08 and OR: 1.10, 95% CI: 0.98–1.23; *P* = 0.11 respectively.

### Publication bias

**Fig. 12:**
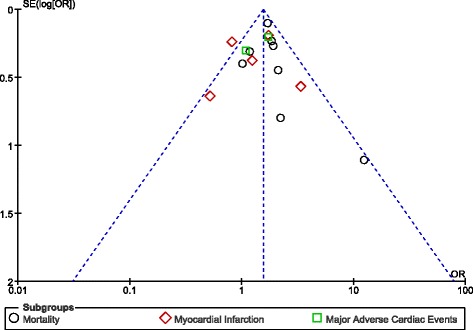
Funnel plot representing publication bias across studies which assessed the short-term outcomes following percutaneous coronary intervention between male and female patients with diabetes mellitus

**Fig. 13:**
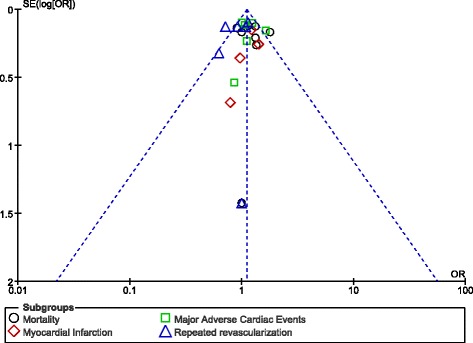
Funnel plot representing publication bias across studies which assessed the long-term outcomes following percutaneous coronary intervention between male and female patients with diabetes mellitus

Based on a visual assessment of the funnel plots which were obtained (Figs. 12 and 13), there has been little evidence of publication bias across the studies that assessed all of these cardiovascular outcomes observed between male and female patients with T2DM following PCI.

## Discussion

A worse prognosis is often associated with patients suffering from T2DM following coronary angioplasty compared to patients without T2DM. However, the fact that whether women or men with diabetes mellitus are at higher risk of suffering from cardiovascular complications is still controversial. Therefore, in this analysis, we aimed to systematically compare the adverse outcomes observed between male and female patients with T2DM following PCI.

The current results showed significantly higher short and long-term mortality and MACEs in female patients with T2DM. This result was more prominent among diabetic patients with acute myocardial infarction. Such a result could have been due to the fact that an increased percentage of co-morbidities such as hypertension and dyslipidemia was observed in female patients with reference to this current analysis. For example, study Blondal2012 [9] consisted of 80.2% versus 91.6% of male and female patients with hypertension respectively, and 62.5% versus 69.7% of male and female patients with dyslipidemia respectively. Other registries also showed women to suffer more co-morbidities than men. In addition, female patients were also older when compared to the male population and this observation was clearly visible at baseline.

Similarly, Insights from a nationwide registry (1997–2010) showed significantly higher in-hospital mortality among women with diabetes when compared to male patients with the same disease following PCI [10]. In addition, at baseline, female patients were older (73.0 versus 66.4 years), majority with hypertension compared to male patients at baseline.

Recently, Liu et al. also showed higher complications to be associated with women (with diabetes mellitus) when compared to men, following coronary angioplasty [11]. In their study, women were older, majority with dyslipidemia compared to men. The Bypass Angioplasty Revascularization Investigation (BARI) 2 Diabetes Trial also supported the result of this current analysis suggesting that higher co-morbidities increased severity of symptoms which were present in these women with T2DM [12]. It should be noted that these women with T2DM also had an advanced age and a longer duration of diabetes mellitus when compared to male patients from the BARI 2 Diabetes Trial. To support the fact that higher co-morbidities which were associated with women might have been responsible for such outcomes, a patient-level pooled analysis of randomized controlled trials showed that among women who underwent PCI with drug eluting stents (DES), chronic kidney disease was associated with a main risk for MACE [13].

Kautzky-Willer et al. showed that women with T2DM had a worse diabetic profile and could achieve therapeutic goals less frequently compared to males with T2DM and suggested more aggressive treatments in women [14]. Moreover, even if Champney et al. concluded that diabetes mellitus was no longer a main risk factor for women who underwent coronary angioplasty, their results showed diabetes mellitus to have a strong association with adverse cardiovascular outcomes in women compared to men when gender and diabetes were to be considered [15].

A meta-analysis of sex-related differences in outcomes following primary PCI for ST segment elevation MI also showed female gender to be associated with significantly higher short-term mortality with OR: 1.10, 95% CI: 1.02–1.18; *P* = 0.02, I^2^ = 83% [16]. However, the result was highly heterogeneous. It should be noted that the analysis consisted of 5832 women with T2DM which was almost comparable to this current analysis. In addition, no significant difference in mid-term mortality was observed with OR: 1.01, 95% CI: 0.99–1.03; *P* = 0.46, I^2^ = 1%.

The ‘smoking paradox’ [17] is another possible reason which might have contributed to a low level of adverse clinical outcomes in these male patients with T2DM following PCI. At baseline, majority of male patients were current smokers when compared to female patients. Study Blondal2012 (30.3% versus 7.00%), study Buja2012 (52.1% versus 16.5%), study Lin2013 (42.3% versus 11.6%), and study Younan2015 (45.0% versus 3.00%) are a few examples that showed a higher percentage of smokers among men. This unexpected phenomenon called the smoking paradox could be another factor contributing to such a result [18, 19].

Recent research has also shown women with T2DM to be at higher risk of adverse events due to the onset mechanism of acute MI, the high burden of cardiac risk factors and conditions which are associated with the procedural success rate following PCI such as bleeding events [20, 21]. Other studies have also shown women to be at a higher risk of developing diffuse small vessel disease which increased their chances of suffering from diabetic cardiomyopathy. In addition, some important biological differences were observed in the content of atherosclerotic plaque in men and women making women to have a more complicated disease or lesion compared to men with the same condition [22].

Also, the decrease or absence of ovarian hormones in menopause women, might further contribute to cardiovascular complications following PCI [23]. Moreover, endothelial dysfunction, functional contraction of the myocardium, platelet aggregations and other regulatory mechanisms might contribute to higher adverse cardiac events in women compared to men with the same clinical conditions [24, 25]. However, other factors should further be investigated.

### Novelty

This research is new because:

- It is among the first few meta-analyses comparing adverse cardiovascular outcomes in male versus female patients with T2DM following PCI.
- This research represents an interesting idea which should be debated in clinical medicine.
- A very low or moderate level of heterogeneity was observed among several of the subgroups analyzing specific outcomes.
- A large number of patients from different corners of the globe were included compared to previously published articles.

### Limitations

Limitations are as follows:

- A limited sample size could affect the results.
- Mainly data which were obtained from observational studies were included in this analysis.
- The endpoints were analyzed using a limited number of studies.
- Different follow up periods in different studies could have affected the results.
- The duration of diabetes mellitus, the length of dual anti-platelet therapy use and the use of other cardiovascular and oral anti hypoglycemic medications as well as the use of insulin therapy could also have had an effect on the outcomes between male and female patients with diabetes mellitus. Unfortunately, the medication profile was limitedly reported in these studies.

## Conclusions

Following PCI, women with T2DM were indeed more susceptible to short and long-term cardiovascular complications compared to male patients with the same chronic disease. Even though this result was more applicable to patients with acute myocardial infarction, the fact that women were older with higher co-morbidities at baseline compared to men, should also not be ignored.

## Abbreviations

MACEs: major adverse cardiac events
MI: myocardial infarction
PCI: percutaneous coronary intervention
T2DM: type 2 diabetes mellitus
